# Supplementary material for: End Sequence Analysis Toolkit (ESAT) expands the extractable information from single-cell RNA-seq data
Source: Genome Res. 2016 Oct;26(10):1397–410. doi: 10.1101/gr.207902.116 (PMC5052061; doi:10.1101/gr.207902.116)
Supplement: Supplemental Material [file supp_gr.207902.116_Supp_Materials.tar.gz › ESAT-esat_v0.1/doc/html/classbroad_1_1core_1_1datastructures_1_1_generic_rooted_tree_3_01_t_01_4.html]

ESAT: broad.core.datastructures.GenericRootedTree< T > Class Reference


ESAT


- Main Page
- Related Pages
- Packages
- Classes
- Files

- Class List
- Class Index
- Class Hierarchy
- Class Members

All Classes Namespaces Files Functions Variables Enumerator Pages

- broad
- core
- datastructures
- GenericRootedTree< T >

Classes |
Public Member Functions |
List of all members

broad.core.datastructures.GenericRootedTree< T > Class Reference

|  |  |
| --- | --- |
| Classes | |
| class | **TreeNode< T >** |
|  | |

|  |  |
| --- | --- |
| Public Member Functions | |
|  | GenericRootedTree (String name, T rootElement) |
|  | |
| TreeNode< T > | getRoot () |
|  | |
| String | getName () |
|  | |
| List< TreeNode< T > > | getNodes (int level) |
|  | |
| List< TreeNode< T > > | getLeaves () |
|  | |
| List< T > | getNodeElements (int level) |
|  | |

## Constructor & Destructor Documentation

|  |  |  |  |
| --- | --- | --- | --- |
| broad.core.datastructures.GenericRootedTree< T >.GenericRootedTree | ( | String | *name*, |
|  |  | T | *rootElement* |
|  | ) |  |  |

## Member Function Documentation

|  |  |  |  |  |
| --- | --- | --- | --- | --- |
| List<TreeNode<T> > broad.core.datastructures.GenericRootedTree< T >.getLeaves | ( |  | ) |  |

|  |  |  |  |  |
| --- | --- | --- | --- | --- |
| String broad.core.datastructures.GenericRootedTree< T >.getName | ( |  | ) |  |

|  |  |  |  |  |  |
| --- | --- | --- | --- | --- | --- |
| List<T> broad.core.datastructures.GenericRootedTree< T >.getNodeElements | ( | int | *level* | ) |  |

|  |  |  |  |  |  |
| --- | --- | --- | --- | --- | --- |
| List<TreeNode<T> > broad.core.datastructures.GenericRootedTree< T >.getNodes | ( | int | *level* | ) |  |

|  |  |  |  |  |
| --- | --- | --- | --- | --- |
| TreeNode<T> broad.core.datastructures.GenericRootedTree< T >.getRoot | ( |  | ) |  |

---

The documentation for this class was generated from the following file:

- java/broad/core/datastructures/GenericRootedTree.java


---

Generated on Fri Aug 15 2014 15:10:51 for ESAT by  

 1.8.7
